# Predicting Early Mortality after Thoracic Endovascular Aneurysm Repair: A Machine Learning-Based Decision Tree Analysis

**DOI:** 10.3400/avd.oa.25-00009

**Published:** 2025-05-23

**Authors:** Masaki Kano, Toshiya Nishibe, Tsuyoshi Iwasa, Seiji Matsuda, Shinobu Akiyama, Toru Iwahashi, Shoji Fukuda, Yusuke Shimahara, Masayasu Nishibe

**Affiliations:** 1 Department of Cardiovascular Surgery, Tokyo Medical University, Tokyo, Japan; 2 Department of Medical Informatics and Management, Hokkaido Information University, Ebetsu, Hokkaido, Japan; 3 Department of Surgery, Eniwa Midorino Clinic, Eniwa, Hokkaido, Japan

**Keywords:** thoracic aortic aneurysm, thoracic endovascular aneurysm repair, mortality, machine learning, decision tree analysis

## Abstract

**Objectives:** Thoracic endovascular aneurysm repair (TEVAR) has revolutionized the treatment of thoracic aortic aneurysms (TAA) by providing a less invasive alternative to open surgery. This study aims to identify risk factors for early mortality following TEVAR for degenerative TAA using a machine learning-based decision tree analysis (DTA).

**Methods:** This retrospective observational study analyzed 79 patients who underwent elective TEVAR to identify predictors of early mortality (within 2 years) using decision tree analysis. The dataset included 36 variables, covering age, sex, nutritional status, comorbidities, inflammation, immune status, and surgical details. The decision tree classifier was developed and validated using Python 3.7 with the scikit-learn toolkit.

**Results:** DTA identified octogenarian status as the strongest predictor of early mortality, followed by poor nutritional status, debranching procedures, and compromised immunity. The model identified 7 terminal nodes, with early mortality risk ranging from 0% to 77.7%. It demonstrated moderate accuracy (65.8%) and high sensitivity (81.0%) but had relatively low specificity (60.3%), effectively identifying high-risk patients.

**Conclusions:** Machine learning-based DTA identified key predictors of early mortality following TEVAR, including octogenarian status, poor nutritional status, compromised immunity, and debranching procedures. The model provides an interpretable risk stratification tool, but its clinical applicability requires further validation.

## Introduction

Thoracic endovascular aneurysm repair (TEVAR) is widely accepted as a minimally invasive alternative to open surgery for thoracic aortic aneurysms (TAA) and is the preferred treatment for high-risk patients due to its low morbidity and reduced short-term mortality.^1)^ However, the overall mortality in high-risk patients remains higher than in normal-risk patients, necessitating careful consideration of life expectancy, surgical risk, and aneurysm-related mortality when selecting the optimal treatment approach for TAA. While several risk factors influencing mortality have been proposed,^2)^ current methods for predicting mortality post-TEVAR are limited, highlighting the need for improved risk stratification.

Machine learning-based decision tree analysis (DTA) offers a highly interpretable and visual approach to identify key risk factors and decision rules in complex medical data.^3)^ This method enables straightforward branching based on specific patient characteristics, making it easy to track and understand. By stratifying patients into different risk groups, decision trees aid in outcome prediction, treatment guidance, and individualized care. Additionally, this approach is adaptable to large datasets and allows for continuous model refinement.

This study aims to identify risk factors of early mortality (within 2 years) after TEVAR for degenerative TAA by developing a predictive model using machine learning-based DTA.

## Materials and Methods

### Patients

This retrospective study included 103 consecutive patients who underwent elective TEVAR for degenerative TAA between January 2013 and December 2021 at the Department of Cardiovascular Surgery, Tokyo Medical University Hospital. Indications for treatment included symptomatic TAA, asymptomatic TAA with a maximum short-axis diameter greater than 55 mm, and rapidly enlarging TAA (greater than 5 mm/6 months), based on the Guidelines for the Diagnosis and Treatment of Aortic Aneurysms and Dissection published by the Japanese Circulation Society.^4)^ Patients with mycotic TAA or a shaggy or heavily calcified aorta were not initially indicated for TEVAR. Patients who had surgery less than 2 years ago or lost to follow-up within 2 years were also excluded.

At the initial visit, all patients were asked if they were willing to provide written informed consent for the use of their clinical data in conference presentations or publications. Procedures were performed in accordance with the ethical standards of the responsible committee (institutional and national) for human experimentation and the Helsinki Declaration of 1975, as revised in 2008. This study was approved by the Clinical Research Committee of Tokyo Medical University Hospital (No. TS2022-0070, 07/15/2022).

The dataset comprised demographic factors (octogenarians, female sex, current smoking, and past smoking); poor nutritional status (defined as geriatric nutritional risk index [GNRI] <98)^5)^; comorbidities (hypertension, diabetes mellitus, dyslipidemia, ischemic heart disease, cerebrovascular disease, chronic obstructive pulmonary disease, chronic kidney disease defined as estimated glomerular filtration rate <60 mL/min/1.73 m^2^, and peripheral artery disease [PAD]); medication (statins, β-blockers, and antiplatelet drugs), aneurysm morphology (aneurysm diameter [≥55 mm vs. <55 mm] and saccular aneurysm); operative data (graft type [expanded polytetrafluoroethylene vs. polyester], multiple devises [defined as ≥2 devices], debranching procedures [one, two, and total debranches], operative endoleaks [Type I, Type II, and Type III]); postoperative complications (cerebral infarction, spinal canal injury, and other serious complications); reinterventions; increased inflammation (defined as C-reactive protein [CRP] >0.3 mg/dL); and compromised immunity (defined as neutrophil-to-lymphocyte ratio [NLR] >3.48).^6)^

The GNRI, which combines albumin and body mass index, was proposed by Bouillanne et al. as an objective nutritional risk index^5)^; it is classified to 4 grades of nutrition-related risk: major risk (GNRI <82), moderate risk (GNRI 82 to <92), low risk (GNRI 92 to ≤98), and no risk (GNRI >98). The GNRI has been shown to be an effective screening tool for assessing nutrition-related morbidity and mortality in a variety of diseases, including heart failure,^7)^ vascular disease,^8,9)^ cancer,^10)^ and other conditions. The NLR, calculated by dividing the absolute neutrophil count by the absolute lymphocyte count, is a useful marker for assessing immune-related clinical outcomes in patients with various diseases.^6,11,12)^ Our previous report determined the cutoff value for the NLR to be 3.48.^6)^

### Surgical procedure

All procedures were performed under general anesthesia with the supervision of certified cardiovascular surgeons (TN, TI) in an angiosuite or an operating room with a portable imaging system. Cervical bypass was performed to ensure an adequate landing zone,^13)^ including one debranching (right subclavian artery [RSCA]-left subclavian artery [LSCA] crossover bypass and left common carotid artery [LCCA]-LSCA bypass), two debranching (RSCA-LCCA-LSCA bypass), and total debranching (carotid artery bypass using bifurcated artificial grafts from the ascending aorta to the RSCA, LCCA, and LSCA).

### Follow-up schedule

Patients were followed up according to the institutional protocol, as reported previously.^6,14)^ Follow-up CT scans were conducted within 1 month, 6 months (optional), and 1 year after TEVAR, with subsequent scans approximately every year thereafter. Endoleaks were routinely assessed by additional CT scans with contrast medium within 1 month, when possible. Thereafter, contrast-enhanced CT scans were performed only if significant sac enlargement >5 mm was detected. Patients were tracked through the database until death or the end of the follow-up period in December 2022, whichever came first. Postoperative outcomes included any causes of death. The patients’ condition (alive or dead) was confirmed through follow-up visits or, for those unable to attend outpatient appointments, by letter or telephone call.

### Statistical analysis

Data were presented as mean ± standard deviation for continuous variables. Prism 9 for MAC OS X (GraphPad Software, La Jolla, CA, USA) was used for statistical analysis. Categorical data were analyzed using the Pearson χ^2^ test or Fisher’s exact probability test. Predictors identified as significant in univariable analysis (p <0.05) were included in the DTA.

### Decision tree analysis

Python 3.7 was used for programming, and the scikit-learn toolkit was employed to derive and validate the decision tree classifier. The classification and Regression Tree (CART) method was applied to predict early mortality,^3)^ with patients who experienced early mortality considered “positive”. The CART method is useful because it can handle both classification and regression tasks, is robust to different data types, and provides interpretable decision trees with pruning to prevent overfitting. Binary trees were constructed to recursively split predictor variables using true/false questions for each variable. The Gini index criterion was used in the CART method.^3)^ The Gini index (impurity) quantifies the likelihood of misclassifying a randomly selected instance, with a lower Gini index indicating a lower probability of misclassification. The Gini index is also preferred over the Entropy index because it is computationally simpler and often leads to faster decision tree construction without significantly compromising accuracy.

To prevent overfitting, the depth of the decision tree was limited to a maximum of 4. Additionally, the minimum sample sizes for internal and terminal nodes were set to 10 and 5, respectively. Branches with fewer samples were not created. These hyperparameters were also optimized using grid search tuning to enhance model performance; grid search is a hyperparameter optimization technique that systematically explores all possible combinations of predefined parameter values to identify the best configuration for the model. Sample weights were applied to account for class imbalances in the target variable. The weight for each class was calculated as the total number of samples divided by the product of the number of classes and the number of samples in each class. Additionally, feature importance was assessed using the Gini impurity criterion, which quantifies each variable’s contribution to reducing classification uncertainty.

The generalization accuracy, sensitivity, and specificity of the model were calculated. Stratified fivefold cross-validation was applied to the test set to assess predictive performance. Patients were randomly divided into five folds, each containing approximately the same number of events. Four folds were used for training the model, while the remaining fold was reserved for validation.

## Results

### Patient characteristics

Of the 103 patients, 79 patients were included in this study, while 24 patients were excluded based on the exclusion criteria; 14 patients who were within 2 years of surgery and 10 patients who were lost to follow-up within 2 years. **Table 1** lists 36 variables including age, gender, nutritional status, comorbidities, inflammation, immune status, and surgical details. Of the included patients, 57 were male (72.2%) and 22 were female (27.8%), with a mean age of 77.1 ± 9.0 years. The majority of patients had cardiovascular risk factors and their associated complications. Commercially available stent grafts were used for treating TAA, with the Gore TAG employed in 42 patients (53.2%, W. L. Gore & Associates, Flagstaff, AZ, USA) and the COOK Zenith in 37 patients (46.8%, Cook, Bloomington, IN, USA). Debranching procedures were performed in 20 patients (25.3%), with one debranching procedure in 9 patients, two debranching procedures in 9 patients, and total debranching procedure in 2 patients.

**Table:** Table 1 Univariable analysis of predictors for early mortality between patients who died within 2 years and those who survived for more than 2 years

Variables	Total (n = 79)	Died (n = 21)	Survived (n = 58)	p value
Age (years)	77.1 ± 7.8	81.0 ± 6.6	75.6 ± 9.4	0.015*
Octogenarian status (≥80 years)	38 (48.1)	15 (71.4)	23 (39.7)	0.021*
Female	22 (27.8)	5 (23.8)	17 (29.3)	0.779
Poor nutritional status (GNRI <101.4)	41 (51.9)	15 (71.4)	26 (44.8)	0.037
Smoking				
Current	18 (22.8)	4 (19.0)	14 (24.1)	0.634
Past	62 (78.5)	13 (61.9)	49 (84.5)	0.989
Concomitant disease				
Hypertension	55 (69.6)	12 (57.1)	43 (74.1)	0.557
Dyslipidemia	20 (25.3)	5 (23.8)	15 (25.9)	0.570
Diabetes mellitus	15 (19.0)	4 (19.0)	11 (19.0)	0.346
Chronic kidney disease	43 (54.4)	9 (42.9)	34 (58.6)	0.214
Chronic obstructive pulmonary disease	10 (12.7)	0 (0)	10 (17.2)	0.055
Cerebrovascular disease	16 (20.3)	4 (19.0)	12 (20.7)	0.873
Carotid artery disease	5 (6.3)	1 (4.8)	4 (6.9)	>0.999
Ischemic heart disease	22 (27.8)	7 (33.3)	15 (25.9)	0.513
PAD	8 (10.1)	5 (23.8)	3 (5.2)	0.028*
Medication				
Antiplatelet drug	39 (49.4)	12 (57.1)	27 (46.6)	0.406
Statin	25 (31.6)	8 (38.1)	17 (29.3)	0.458
β-blocker	38 (48.1)	11 (52.4)	27 (46.6)	0.547
Aneurysm anatomy				
Aneurysm diameter	53.2 ± 9.9	56.1 ± 11.8	51.9 ± 8.7	0.103
≥55 mm	30 (38.0)	11 (52.4%)	19 (32.8)	0.171
Saccular	34 (43.0)	8 (38.1)	26 (44.8)	0.619
Operative data				
Graft type				
Expanded polytetrafluoroethylene	42 (53.2)	12 (57.1)	30 (51.7)	0.670
Polyester	37 (56.8)	9 (42.9)	28 (48.3)	Reference
Multiple devices (≥2 devices)	37 (46.3)	11 (52.4)	26 (44.8)	0.552
Debranching procedures	20 (25.3)	9 (42.9)	11 (19.0)	0.031*
One	9 (11.4)	3 (14.3)	6 (10.3)	0.693
Two	9 (11.4)	4 (19.0)	5 (8.6)	0.236
Total	2 (2.5)	2 (9.5)	0 (0)	0.682
Operative endoleak	14 (17.7)	5 (23.8)	9 (15.5)	0.501
Type I	8 (10.1)	3 (14.3)	5 (8.6)	0.432
Type II	5 (6.3)	2 (9.5)	3 (5.2)	0.805
Type III	1 (1.2)	0 (0)	1 (1.7)	>0.999
Postoperative complication	8 (10.1)	3 (14.3)	5 (8.6)	>0.999
Cerebral infarction	3 (3.8)	2 (9.5)	1 (1.7)	0.171
Spinal canal injury	2 (2.5)	0 (0)	2 (3.4)	>0.999
Other serious complications	3 (3.8)	1 (4.8)	2 (3.4)	>0.999
Reintervention	10 (12.7)	1 (4.8)	9 (15.5)	>0.999
Increased inflammation (CRP >0.3 (mg/dL)	68 (86.1)	19 (90.5)	49 (84.5)	0.718
Compromised immunity (NLR >3.48)	24 (30.4)	10 (47.6)	14 (24.1)	<0.001*

Unless indicated otherwise, data are presented as mean ± SD or n (%).

*Significant.

GNRI: geriatric nutritional risk index; PAD: peripheral artery kidney disease; CRP: C-reactive protein; NLR: neutrophil-to-lymphocyte ratio

### Follow-up

Of the 79 patients, 58 (73.4%) survived for more than 2 years, while 21 (26.6%) died within 2 years. Causes of death included pneumonia (7), cancer (2), interstitial pneumonia (2), sepsis (2), type A aortic dissection (1), acute myocardial infarction (1), peritonitis (1), pulmonary embolism (1), cerebral hemorrhage (1), and three deaths of unknown but not related to aneurysm. No aneurysm-related death occurred.

### Risk factors for early mortality

Significant differences were observed in five variables between patients who survived for more than 2 years and those who died within 2 years: octogenarian status (p = 0.021), poor nutritional status (p = 0.037), compromised immunity (p <0.001), PAD (p = 0.028), and debranching procedures (p = 0.031) (**Table 1**). No significant differences were found in the other variables between these two groups.

### Decision tree analysis

**Figure 1:**
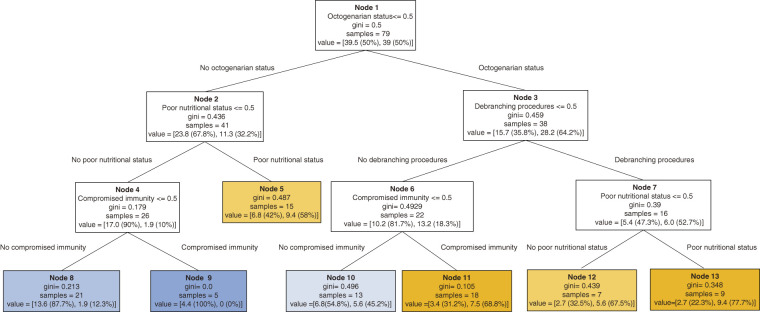
Fig. 1 A decision tree generated using the classification and regression tree (CART) methodology to illustrate the hierarchical relationship between early mortality and five variables identified in the univariable analysis, including octogenarians, poor nutritional status, compromised immunity, peripheral artery disease, and debranching procedures. For example, the first node “octogenarian status <= 0.5” means that if the patient is not “octogenarian status (value = 1)”, it branches to the left (true), and if the patient is “octogenarian status”, it branches to the right (false). Blue nodes represent higher survival likelihood, while orange nodes indicate higher mortality risk.

Using the CART technique, five variables identified in the univariable analysis, including octogenarian status, poor nutritional status, compromised immunity, PAD, and debranching procedures, were able to segment the data according to the risk of early mortality. The decision tree is shown in **Fig. 1**. The root node (**Node 1**), representing the most significant variable, was octogenarian status. Although this root variable alone cannot determine truth/false outcomes, it is the first variable evaluated in the decision process. The next significant split **(Node 2**) was poor nutritional status. Subsequent branches revealed the compounded risk of debranching procedures (**Node 3**), compromised immunity (**Node 4**), highlighting a complex interplay of factors influencing early mortality. PAD was not included as a node in the decision tree. The decision tree identified seven terminal nodes, with the risk of early mortality ranging from 0% (**Node 9**) to 77.7% (**Node 13**). Four of the terminal nodes identified high-risk groups with early mortality probabilities of 58.0% (**Node 5**), 67.5% (**Node 12**), 68.8% (**Node 11**), and 77.7% (**Node 13**). Conversely, three other terminal nodes identified low risk groups of patients with early mortality probabilities of 0% (**Node 9**), 12.3% (**Node 8**), and 45.2% (**Node 10**).

This decision tree model achieved an accuracy of 65.8%, sensitivity of 81.0%, and specificity of 60.3%. Additionally, the model demonstrated a generalization accuracy of 62.2 ± 11.3%, sensitivity of 71.0 ± 32.9%, and specificity of 58.6 ± 23.3% for the training sets.

### Feature importance

**Figure 2:**
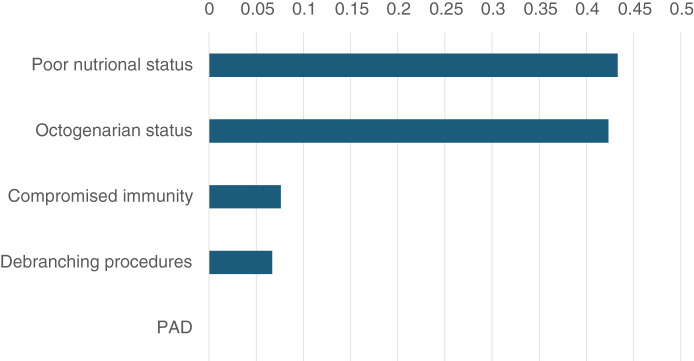
Fig. 2 Key predictors of early mortality identified by the decision tree model. PAD: peripheral artery disease

Two important predictors were identified as poor nutritional status, defined by GNRI <98, and octogenarian status, with an importance score of 0.4333 and 0.4234, respectively (**Fig. 2**). Other predictors included compromised immunity, defined as NLR >3.48, (importance score: 0.0762), and debranching procedures (importance score: 0.0670), but not PAD (importance score: <0.0000).

## Discussion

This study used machine learning-based DTA to identify key risk factors associated with early mortality in patients undergoing TEVAR for degenerative TAA. A combination of patient characteristics and procedural factors, including older age (octogenarian status), poor nutritional status, compromised immunity, and concomitant debranching procedures, were strongly associated with an increased risk of early mortality within 2 years postoperatively. The decision tree model provided an interpretable framework for assessing early mortality risk with moderate predictive accuracy.

The structure and adaptability of DTA make it a particularly powerful tool in clinical decision-making.^15)^ DTA accommodates both categorical and continuous data, providing a flexible model structure that effectively manages missing values.^3)^ The visual decision tree format improves communication between physicians and patients, as the branching paths clearly illustrate how each factor contributes to the overall risk profile.^16)^ This transparency not only aids clinicians in coordinating care but also supports shared decision-making by enabling patients to better understand their own risk factors.

The decision tree demonstrated that, among the identified factors, older age (octogenarian status) was the most significant predictor of early mortality following TEVAR. However, it highlighted that older age alone is not a decisive factor, rather, it interacts with other risk factors, such as poor nutritional status and compromised immunity. Poor nutritional status, indicated by a low GNRI, was a critical branch in the decision tree, emphasizing the importance of nutrition in surgical outcomes. Previous studies have shown that poor nutrition is associated with adverse postoperative outcomes after TEVAR, highlighting the need for nutritional assessment and potential interventions in high-risk patients.^17,18)^ Compromised immunity, measured by an elevated NLR, also emerged as a key determinant of early mortality after TEVAR.^6)^ Elevated NLR has been linked to poor outcomes in various diseases and surgical procedures.^6,11,12)^ An increased NLR may reflect underlying immune dysregulation, which could lead to susceptibility to infection and slower recovery, thereby increasing the risk of mortality.^19)^

Concomitant debranching procedures were also identified as a significant risk factor influencing early mortality in older patients. Patients requiring complex debranching likely represent a subgroup with extensive aneurysmal disease or anatomical challenges that complicate TEVAR.^13)^ These findings suggest that technical complexity may inherently increase morbidity and mortality, potentially due to the extended procedural time, risk of vascular injury, or increased perioperative complications associated with debranching. Careful patient selection and thorough preoperative planning are crucial for optimizing outcomes. Further studies are needed to explore whether procedural modifications or supportive perioperative interventions could improve survival in this patient subgroup.

The decision tree model achieved a moderate accuracy of 65.8% and high sensitivity of 81.0%, indicating its effectiveness in identifying patients at risk of early mortality. Although specificity was lower, the model can still serve as a valuable screening tool in clinical practice as it effectively captures high-risk patients. The interpretability of the decision tree allows clinicians to use its structure to pinpoint high-risk individuals and adjust their preoperative or postoperative care plans accordingly. However, due to the model’s limitations, such as low specificity and generalizability, it is intended to augment, rather than replace, clinical judgment in the decision-making process.

### Study limitations and future directions

Several limitations should be acknowledged. First, it was conducted retrospectively at a single institution with a relatively small sample size, which may limit the generalizability of the findings. While the decision tree provided valuable insights into early mortality risk factors, machine learning models can be sensitive to overfitting, especially in studies with limited data. Second, due to institutional limitations, we could not access an independent dataset. To address this, we performed stratified fivefold cross-validation, which provides an internal assessment of model generalizability. Theoretically, fivefold cross-validation can serve as a substitute for external validation by repeatedly partitioning the dataset into training and validation sets, ensuring robust performance estimation. This approach also helps mitigate overfitting and provides a more comprehensive assessment of the model’s predictive ability. Third, the moderate accuracy and low specificity observed also suggest that other machine learning techniques, or the incorporation of additional clinical and biomarker data, may enhance predictive performance, in addition to the need for larger, prospective studies. Future research should validate these findings in larger, multicenter cohorts and explore the integration of additional variables, such as biomarkers of inflammation and detailed anatomical characteristics, to improve model precision. Finally, prospective studies could investigate the impact of targeted interventions, such as nutritional support or immunomodulatory therapies, on the outcomes of high-risk patients identified through this model.

## Conclusions

Machine learning-based DTA identified key predictors of early mortality following TEVAR, including octogenarian status, poor nutritional status, compromised immunity, and debranching procedures. The model provides an interpretable risk stratification tool, but its clinical applicability requires further validation. Future large-scale research should integrate additional variables and external datasets to enhance predictive performance.

## Declarations

### Funding

There was no funding for this research.

### Disclosure statement

The authors report no actual or potential conflict of interest in relation to this article.

### Author contributions

Study conception: MK, TN

Data collection: MK, TN, SA, TI2, SF, YS

Analysis: MK, TN, TI1, SM

Investigation: MK, TN

Manuscript preparation: MK, TN, MN

Critical review and revision: all authors

Final approval of the article: all authors

Accountability for all aspects of the work: all authors.
